# The promise of one health for improved soil and food security in Papua New Guinea

**DOI:** 10.1016/j.onehlt.2026.101341

**Published:** 2026-01-29

**Authors:** Tom Swan, Josephine Saul Maora, Barbara Pamphilon, Damien Field

**Affiliations:** a School of Life and Environmental Sciences, Sydney Institute of Agriculture, The University of Sydney, Sydney, New South Wales, Australia; b Family Farm Teams Country Leader, Port Moresby, Papua New Guinea; c Centre for Sustainable Communities, University of Canberra, Australian Capital Territory, Canberra, Australia

**Keywords:** One health, Soil security, Food security, Stunting, Malnutrition, Soil health restoration, Mangrove rehabilitation, Papua New Guinea

## Abstract

Papua New Guinea (PNG) faces a critical food security crisis, with nearly half of children under five experiencing stunting—more than twice the global average. Combined with high rates of wasting and overweight, this reflects the country's ‘double burden of malnutrition’. Furthermore, public health and environmental pressures in PNG are intensifying, particularly as climate change reduces crop yields, nutrient density and ecosystem stability. Addressing these interconnected challenges demands integrated strategies that strengthen both food and soil security. This article applies the One Health framework to PNG's social-ecological system by examining two interventions: soil health restoration and mangrove ecosystem rehabilitation. These interventions enhance landscape resilience, restore degraded soils and sustain protein-rich food sources. Our novel PNG One Health (*Wanpela Helt*) framework—a culturally grounded approach—emphasises strong soil, strong food and strong community (*strongpela giraun, strongpela kaikai, strongpela komuniti*), with family, community-based approaches and gender equity as cornerstones for sustainable development.

## 1 Introduction

**Fig. 1:**
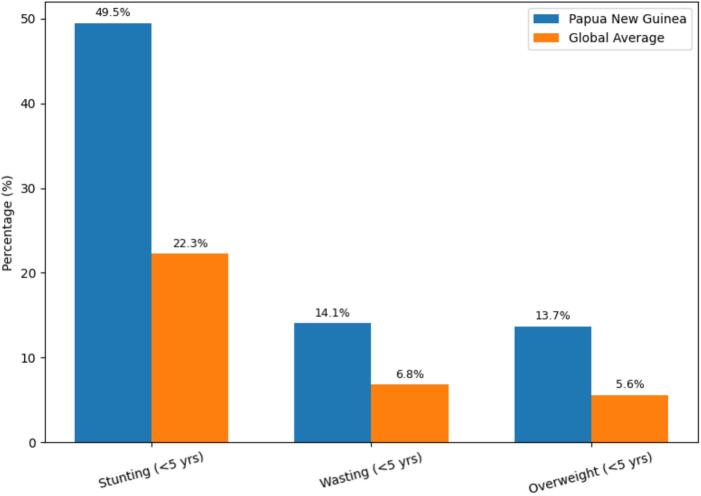
Malnutrition indicator (stunting, wasting, and overweight) estimates among children under five: comparison between Papua New Guinea (blue) and global averages (orange). Sources: [11,12]. (For interpretation of the references to colour in this figure legend, the reader is referred to the web version of this article.)

Papua New Guinea (PNG), a nation of approximately 11 million people, faces intersecting environmental and climate-related threats that are magnified by socio-economic and political fragility [1]. Widespread poverty, inadequate infrastructure and dependence on subsistence agriculture further constrain adaptive capacity as climate change weakens food systems and heightens vulnerability to food insecurity. Food insecurity, defined as “a situation when people lack secure access to sufficient, safe, and nutritious food for normal growth, development, and an active, healthy life” [2]—is common in PNG [3,4]. Food insecurity is most acute in rural areas, where approximately 86% of the population resides and produce 96% of the nation's food [5]. Diets in rural areas are dominated by carbohydrate-rich staples such as root crops, sago, and bananas, with limited access to protein-rich foods [6]. This nutritional imbalance compounded by climate-induced food shortages [7] and inadequate protein and energy intake among children under five, is most evident in the high rates of stunting (49.5%), wasting (14.1%), and overweight (13.7%)—all exceeding global averages (Fig. 1). In adults, poor nutritional status is increasingly reflected in impaired metabolic and cardiovascular health, reduced economic productivity, and other lifelong consequences of malnutrition [8]. This reveals the ‘double burden of malnutrition’ in PNG: chronic undernutrition alongside rising obesity and diet-related diseases [9]. Evidence indicates that interventions to reduce stunting in PNG should begin before and during pregnancy, as well as postnatally [10]. Yet, such interventions alone cannot address the root causes of malnutrition which include poverty, poor diet diversity, inadequate health services and soil degradation.

Enhancing food security in PNG also depends on advancing soil security—the maintenance of soils to sustain life on Earth [13]. Soil security, a critical yet underappreciated concept, is closely linked to food security [14] and is also fundamental for improving the resilience and nutritional quality of PNG's food systems. Achieving the much-needed progress toward food and soil security in PNG requires integrated, holistic strategies grounded in One Health principles.

### 1.1 One health and the PNG socio-ecological systems

One Health approaches in PNG [e.g., 15] offer valuable strategies for addressing interconnected challenges such as food and soil security [16,17]. The Lancet One Health Commission [18] emphasises that advancing One Health requires integrating social-ecological systems (SES) into its framework. Social-ecological systems are defined as diverse, intricate, and dynamic inter-relations and mutual dependencies that exist both within and between the ecological systems of the natural world and the social, cultural, political, and economic systems shaped by human societies [19].

**Fig. 2:**
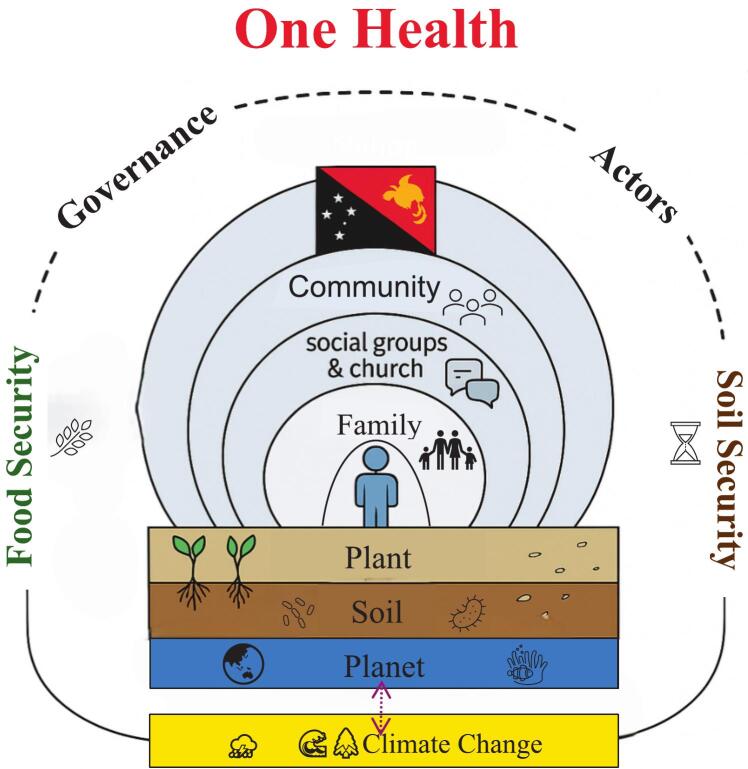
Proposed One Health in social-ecological systems (OHSES) model, integrating food and soil security for PNG. Climate change disrupts ecological processes (e.g., rainfall, temperature, sea-level rise) and social systems (e.g., food availability, economic stability), interacting with governance and socio-political structures that shape the pace and impacts of climate change.

Building on [20] One Health (OHSES) integrated perspective, Fig. 2 provides an original PNG example. It shows nested layers: an individual at the centre, surrounded by family, social groups and church, community, and nation—all embedded within environmental components (planet, soil and plant). Climate change exerts systemic pressure, disrupting ecological processes (e.g., altered rainfall patterns, rising temperatures and sea-level rise) and social systems (e.g., food availability, economic stability and poverty). These disruptions interact with governance, actors and socio-political-economic systems, which in turn influence the pace of climate change—for instance, through the presence or absence of policies aimed at climate mitigation and/or adaptation. This dynamic creates a feedback loop, where climate impacts and societal responses continuously shape one another. While climate change disrupts ecological and social systems, policy responses and governance structures influence the trajectory of these impacts, all influencing food and soil security.

Fig. 2 highlights the complexity of PNG's climate–society feedback loops under a OHSES lens, highlighting the need for interventions that span both ecological and social dimensions, as discussed in the next section.

## 2 Practical interventions for food and soil security in Papua New Guinea

Two illustrative and complementary interventions for enhancing PNG food and soil security can be seen in the rehabilitation of mangrove ecosystems for coastal food sources and the restoration of soil health for food crops. Both approaches strengthen climate resilience by improving soil health (specifically soil condition, a key dimension of soil security [16]) and supporting food security. Healthy soils sequester carbon, reduce erosion and improve nutritionally rich food crops, while mangroves protect coastlines, limit saltwater intrusion and provide habitats for fisheries that ensure animal protein-rich food sources.

By improving soil health and rehabilitating mangroves, these interventions collectively strengthen food security, support animal well-being (e.g., habitat provision and benthic food availability), and reduce human exposure to contaminated or diseased aquatic species—reinforcing the interconnected environment–animal–human triad central to One Health.

### 2.1 Mangrove ecosystem rehabilitation

Mangrove ecosystems are increasingly degraded globally and in PNG extensive areas of the coastline are now classified as endangered [21]. This degradation threatens the vital social-ecological services that mangroves provide. Mangrove restoration delivers multiple benefits: shielding coastlines from storms and erosion, sequestering carbon, and supporting livelihoods [22]—particularly through fisheries, as mangroves provide essential nursery habitats [23] that underpin protein sources for communities.

Successful mangrove restoration relies on long-term monitoring, community engagement, integration of Indigenous knowledge, and capacity building within local institutions [24]. In PNG, mangrove restoration initiatives such as the MARSH Project and Women in Mangrove Management (WIMA), illustrate strong community engagement and positive outcomes, strengthening soil and food security. However, sustaining these efforts can be difficult when resources for monitoring and technical support and effective policy implementation are limited [25] or when donor priorities shift, as occurred during MARSH's implementation. Addressing these barriers is critical for scaling and sustaining rehabilitation efforts and can be advanced through strengthened long-term financing, enhanced local monitoring and technical capacity, and the integration of rehabilitation priorities into stable national policies—measures that also help safeguard fish health and animal well-being as restored habitats recover.

### 2.2 Soil health restoration

As subsistence farming is the nutritional mainstay for rural communities, healthy soils are the foundation of PNG's food systems. When soils degrade, crop yields and quality decline, reducing dietary diversity and increasing reliance on imported foods, in turn undermining national food security. Sustainable soil management strategies in PNG [26] include agroforestry (e.g., intercropping and shading), nutrient recycling (e.g., composting and mulching), and erosion control (e.g., contour planting and perennial grasses). Perennial grasses, such as vetiver have been successfully trialled in PNG for soil stabilization, including in saline conditions [27], offering a scalable solution for protecting unstable slopes and reducing erosion. This is crucial for ensuring sustainable food production and protecting soil and land resources, particularly relevant in geologically unstable highland terrains and erosion-prone coastal regions.

Soil health interventions in PNG face significant barriers. Implementing practices such as agroforestry, composting, and erosion control requires access to organic inputs, seedlings, and tools—resources that are often unaffordable and/or scarce. Limited extension services further constrain adoption. In the absence of widespread economic incentives such as soil carbon credits, farmers often prioritize short-term economic gains over practices that promote long-term soil health, such as intercropping and nutrient cycling. Commercial demand for crops such as oil palm and balsa has driven shifts from agroforestry to monocultures in many parts of PNG and other tropical countries, degrading soil condition and resilience. Achieving lasting soil health demands One Health informed evidence-based integrative approaches, which are presented in the following sections.

### 2.3 Family-based food security

Given PNG's food security challenges and limited access to agricultural extension services, several family-focused agricultural development programs are supporting semi-subsistence farmers to transition to planned farming and strengthen sustainable livelihoods. The PNG-wide Family Farm Teams (FFT) program is one such initiative (see also EU-STREIT PNG and CARE International). Through experiential learning workshops, FFT strengthen agricultural and financial capacity through farm mapping, seasonal planning for nutrition and income, and shared responsibilities across farm and family roles, while promoting equitable decision-making. This family-centred approach aligns closely with One Health by recognising the interconnected environment–animal–human triad and by emphasizing strengths-based cooperation to improve food and soil security and broader community well-being.

FFT and other programs in PNG recognise the inequitable gendered division of agricultural labour—where men typically manage cash crops and control the higher profits they generate, while women tend food crops and retain only the much smaller earnings [28]. Such disparities can result in exclusion, conflict, and gender-based violence. Shared farm planning within FFT helps address these imbalances through joint decision-making and a more equitable distribution of labour and benefits, demonstrating that gender equity is essential because unequal control of income and labour influences soil management, food security, and health—important facets of One Health.

While community- and family-based approaches offer strong potential for resilience, their scalability remains challenging. Training and capacity building require considerable time and investment—long-term monitoring is essential—and government or donor support is often short-term and/or limited. These constraints underscore the need for realistic planning and sustained, long-term institutional commitment to ensure continuity and lasting impact.

As the next section illustrates, our One Health framework builds on insights from these community-based programs.

## 3 The PNG one health framework

In PNG, knowledge exchange primarily takes place in social and *wantok* networks. In Tok Pisin, the national lingua franca, *wantok* translates to ‘one talk’ and describes a reciprocal exchange of support and assets among direct clan [29]. Social and *wantok* networks can facilitate the exchange of food, harvests, labour, and soil management advice [30], while also providing financial and social support, thereby strengthening community cohesion.

**Fig. 3:**
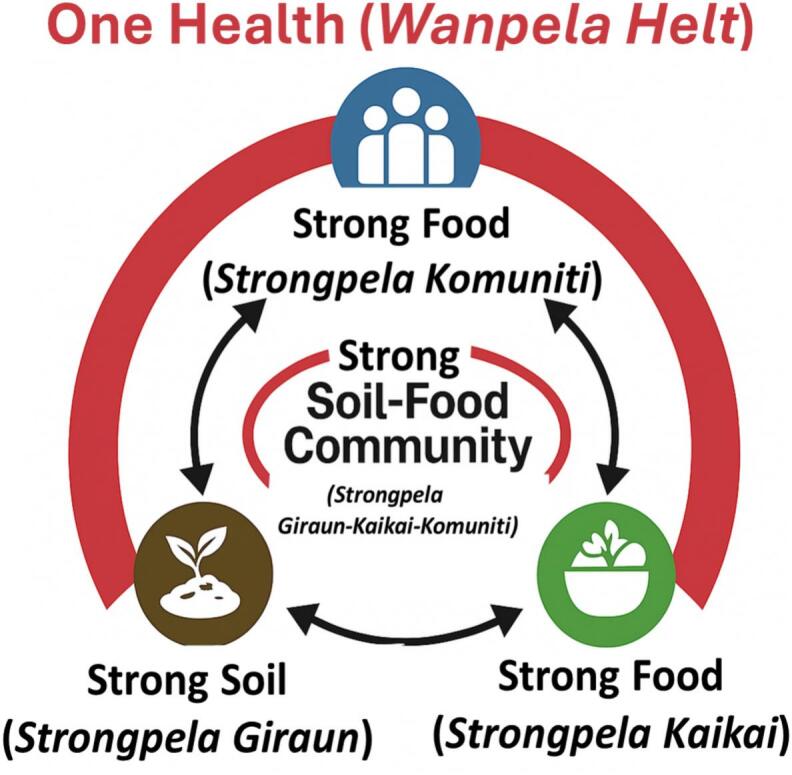
The Soil-Food-Community (*Giraun-Kaikai-Komuniti*) triad illustrates the interconnected relationships of under a One Health (*Wanpela Helt*) framework. Tok Pisin translations are italicised and displayed in brackets. The word strong (*strongpela*) is used intentionally: in Tok Pisin, *strongpela* extends beyond physical strength to encompass resilience, authority, and responsibility. We apply strong to soil, food and community to signify resilience, sustainability and interconnectedness as foundations for adaptation.

Our novel One Health (*Wanpela Helt*) conceptual framework (Fig. 3), builds on the strengths of the *wantok* system by emphasizing unity and collective well-being through the integration of soil security, food security, and community strength. The Soil–Food–Community (*Giraun-Kaikai-Komuniti*) triad illustrates this interdependence: strong soils support strong food systems, which in turn sustain resilient and strong communities. This triad offers a holistic vision for climate resilience, where social cohesion and knowledge sharing are as important as ecological interventions.

To operationalize this framework, indicators could include existing food security assessments—such as those produced by the PNG Food Security Simulator [31]—alongside soil security assessments [13], and measures of community cohesion or social harmony from community leaders (e.g., cultural, church, school, local government) or proxies like gender-based violence. Such components could be integrated into a composite *Wanpela Helt* score for a given area, with safeguards to protect data privacy.

## 4 Conclusion

By applying a One Health framework through a SES lens (OHSES), we have revealed how interconnected feedback loops between climate and society shape both food and soil security in PNG. Our two intervention examples, the restoration of soil health and the rehabilitation of mangrove ecosystems, show the interdependence of nutrition support, ecosystem stability and community resilience.

We recommend adopting this more holistic One Health (*Wanpela Helt*) framework, which explicitly positions strong soils as a foundation for resilient and sustainable agri-food systems. The ‘double burden of malnutrition’ in PNG highlights the urgent need for integrated, climate-resilient strategies to strengthen both food and soil security. Interventions and strategies should therefore be aligned under a *Wanpela Helt* framework, with a focus on building strong soils, strong food systems, and strong communities.

## Declaration of generative AI and AI-assisted technologies in the manuscript preparation process

During the preparation of this work the TS used Microsoft Copilot to help rephrase sentences for clarity. After using this tool/service, TS reviewed and edited the content as needed and takes full responsibility for the content of the published article.

## CRediT authorship contribution statement

**Tom Swan:** Writing – review & editing, Writing – original draft, Visualization, Validation, Software, Resources, Project administration, Methodology, Investigation, Formal analysis, Data curation, Conceptualization. **Josephine Saul Maora:** Writing – review & editing, Writing – original draft, Visualization, Conceptualization. **Barbara Pamphilon:** Writing – review & editing, Writing – original draft, Visualization, Supervision, Investigation, Conceptualization. **Damien Field:** Writing – review & editing, Writing – original draft, Project administration, Funding acquisition.

## Funding

This study is supported by the 10.13039/501100000974Australian Centre for International Agricultural Research project ‘SLAM/2019/109’ Optimising soil management and health in Papua New Guinea integrated cocoa farming systems (Phase 2).

## Declaration of competing interest

The authors declare that they have no known competing financial interests or personal relationships that could have appeared to influence the work reported in this paper.

## Data Availability

Data that was used to create Figure 1 is publicy available from sources listed in the Figure caption.
